# CD1: A Singed Cat of the Three Antigen Presentation Systems

**DOI:** 10.1007/s00005-017-0461-y

**Published:** 2017-04-06

**Authors:** Radoslaw Kaczmarek, Mariola Pasciak, Katarzyna Szymczak-Kulus, Marcin Czerwinski

**Affiliations:** 1 Laboratory of Glycoconjugate Immunochemistry, Hirszfeld Institute of Immunology and Experimental Therapy, Polish Academy of Sciences, Wrocław, Poland; 2 Laboratory of Medical Microbiology, Hirszfeld Institute of Immunology and Experimental Therapy, Polish Academy of Sciences, Wrocław, Poland; 3 Faculty of Physiotherapy and Physical Education, Opole University of Technology, Opole, Poland

**Keywords:** CD1 proteins, Antigen presentation, iNKT cells, Glycosphingolipids

## Abstract

Contrary to general view that the MHC Class I and II are the kapellmeisters of recognition and response to antigens, there is another big player in that part of immunity, represented by CD1 glycoproteins. In contrast to MHC Class I or II, which present peptides, CD1 molecules present lipids. Humans express five CD1 proteins (CD1a-e), four of which (CD1a-d) are trafficked to the cell surface, where they may display lipid antigens to T-cell receptors. This interaction may lead to both non-cognate and cognate T cell help to B cells, the latter eliciting anti-lipid antibody response. All CD1 proteins can bind a broad range of structurally different exogenous and endogenous lipids, but each shows a preference to one or more lipid classes. This unorthodox binding behavior is the result of elaborate architectures of CD1 binding clefts and distinct intracellular trafficking routes. Together, these features make CD1 system a versatile player in immune response, sitting at the crossroads of innate and adaptive immunity. While CD1 system may be involved in numerous infectious, inflammatory, and autoimmune diseases, its involvement may lead to opposite outcomes depending on different pathologies. Despite these ambiguities and complexity, CD1 system draws growing attention and continues to show glimmers of therapeutic potential. In this review, we summarize the current knowledge about CD1 proteins, their structures, lipid-binding profiles, and roles in immunity, and evaluate the role of CD1 proteins in eliciting humoral immune response.

## Introduction

Major histocompatibility complex (MHC) proteins play an essential role in gnathostome immune system by presenting antigens to T cells. The mainstream part of that mechanism involves presentation of peptide antigens in two possible ways depending on their origin. Proteins derived from phagocytosed pathogens (e.g., from bacteria or fungi) are degraded in the lysosomes: the remnant peptides may be captured by the MHC Class II proteins and trafficked to the cell surface to engage helper T cells. Endogenous proteins, both self and foreign, e.g., viral or derived from intracellular bacteria, undergo degradation in proteasomes, in which case the resulting peptides are carried to the cell surface by MHC Class I. The displayed fragments are then probed by cytotoxic T cells (Neefjes and Ovaa 2013).

However, self-versus-foreign discrimination is not limited to processing and presentation of proteins, but involves lipids as well. Lipid antigens are presented by a distinct family of MHC Class I-like proteins, named CD1 (Table 1). Humans express five isoforms of CD1 (CD1a-CD1e), in contrast to muroids, which express only CD1d isotype (Barral and Brenner 2007), and ruminants, which express all but CD1c (Van Rhijn et al. 2006). CD1d was previously reported to be missing in ruminants too (Van Rhijn et al. 2006; Looringh van Beeck et al. 2009), but recently, it has been shown that the bovine CD1D gene is expressed and the protein structure has been solved (Nguyen et al. 2013; Wang et al. 2012). Two chicken CD1 genes identified so far do not match to any of the mammalian isoforms, and are, therefore, named CD1.1 and CD1.2 (Miller et al. 2005; Salomonsen et al. 2005). Based on the amino-acid sequence, mammalian CD1 proteins have been classified into three groups: CD1a-c belong to group 1 and present lipid antigens to clonally diverse T cells that mediate adaptive immunity, while CD1d proteins make up group 2 and present antigens to natural killer T cells (NKT) (Cerundolo et al. 2009). A subset of these cells expresses an invariant T-cell receptor (TCR) *α*-chain and is, therefore, called invariant NKT cells (iNKT) (Salio et al. 2014). Group 3 includes only CD1e, which in contrast to CD1a-d is not expressed on the cell surface, but serves as a soluble lipid transfer protein in the endolysosomal network. Thus, some authors skip it altogether when writing about CD1 as an antigen presentation system (Ly and Moody 2014).

**Table 1:** Contrasting features of MHC Class I, MHC Class II, and CD1 antigen presentation systems

Feature	MHC Class I	MHC Class II	CD1
Recognized ligands	Peptides	Peptides	Lipids
Ligand processing	Peptides derived from proteasomal degradation of proteins	Peptides derived from lysosomal degradation of proteins	Usually not required
Ligand origin	Endogenous	Exogenous	Exogenous or endogenous
Ligand size and shape	8–9 amino acids long peptides	14–20 amino acids long peptides	Different lipid types of varying shapes and sizes
Binding site structure	Closed-ended groove	Open-ended groove	Larger clefts of varying shapes and sizes, from dead-end single-pass tunnels to riddled maze-like structures
Expression	Most cell types	Antigen-presenting cells	CD1a-c mostly in antigen-presenting cells, CD1d more widely
Degree of polymorphism	Highly polymorphic	Highly polymorphic	Non-polymorphic
Degree of polygenicity	Highly polygenic	Highly polygenic	Non-polygenic (in humans)
Responding T cells	CD8^+^	CD4^+^	*αβ, γδ*, CD4^+^, CD8^+^ or double-negative (CD4^−^CD8^−^), iNKT, NKT type II

Studies on CD1 system in general, and CD1d-iNKT aspect in particular, have been plagued by puzzling and sometimes conflicting reports. Sitting at the crossroads of innate and adaptive immunity, CD1 system contains attractive therapeutic targets, but its complexity and ambiguous roles delay its flourish. Yet, as our understanding of the system improves, it continues to show glimmers of therapeutic potential.

In this review, we summarize the current knowledge about CD1 proteins, their structures, lipid-binding profiles, and roles in immunity, and we attempt to evaluate the role of CD1 proteins in eliciting humoral immune response.

## Exogenous Lipids Presented by CD1 Molecules

**Fig. 1:**
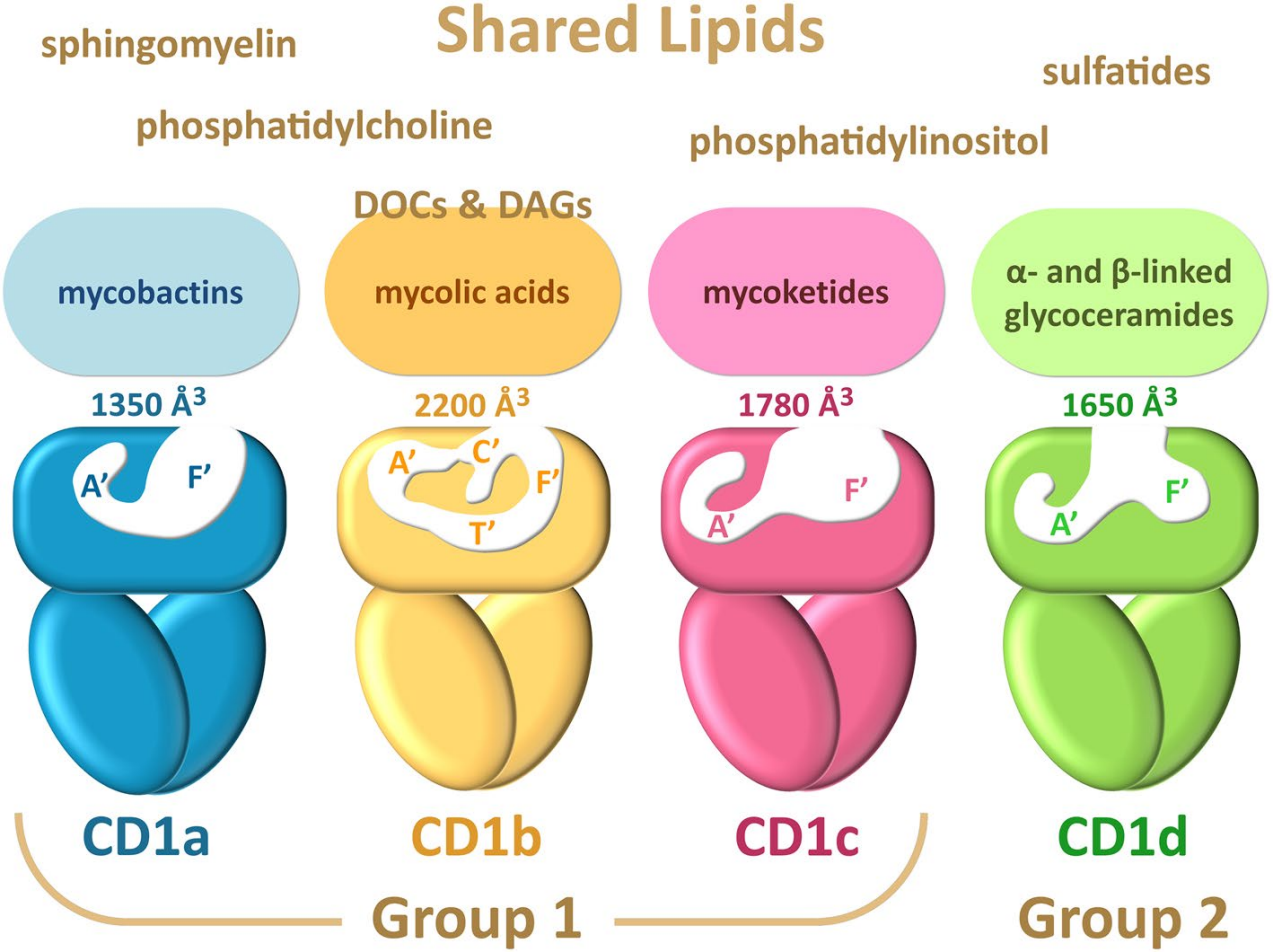
CD1 isoforms show different architectures of their binding clefts, which determine their lipid-binding repertoire. Although each isoform reveals specific binding profile, many (mostly endogenous) lipids can be presented by all CD1 molecules. CD1b utilizes diacylglycerols (DAGs) and deoxyceramides (DOCs) as scaffold lipids when presenting small exogenous lipids, so they are depicted at the interface between shared and CD1b unique lipid repertoire (adapted from Adams 2014)

The ability of CD1 molecules to recognize lipid antigens and present them to specialized subsets of T cells was discovered by studying the immune response to *Mycobacterium tuberculosis* antigens (Beckman et al. 1994). The first described lipid antigens recognized by CD1 system were *α*-branched and *β*-hydroxylated long chain fatty acids called mycolic acids, presented by CD1b (Porcelli et al. 1992). Many more microbial species that engage CD1 system have been identified, including *Sphingomonas, Borrelia*, proteobacteria, fungi and protozoa such as *Leishmania* (Tsuji 2006) (Table 2). CD1 isoforms show different and in some cases overlapping binding profiles, which are related to the architecture of their binding grooves (de Jong et al. 2007) and different trafficking routes through subcellular compartments, causing each isoform to encounter a different set of lipid species *en route* to the cell surface (Lawton and Kronenberg 2004) (Fig. 1). CD1a and (to a lesser extent) CD1c predominantly follow early endosomal pathways and recycle to the cell surface, while CD1b and CD1d efficiently proceed to late endosomes and lysosomes before resurfacing (Salamero et al. 2001; Van Rhijn et al. 2009). This difference may hint at how rodents could afford deleting their group 1 CD1 and partially explain the capacity of CD1d to compensate for the loss (Dascher and Brenner 2003). All CD1 molecules are heavily glycosylated type I integral membrane proteins, and each comprises extracellular *α*1 and *α*2 domains (which bind antigens), and *α*3 domain, located closer to the membrane and non-covalently associated with the light chain of *β*-microglobulin. *α*3 domain and *β*-microglobulin are both members of the immunoglobulin superfamily (Pei et al. 2012). CD1 molecules are structurally similar to MHC Class I proteins, but their binding grooves are larger and lined with hydrophobic amino-acid residues, which reflects the specialization to bind lipids. While anchoring within the CD1 proteins, the hydrophobic parts of antigens are buried deep inside the groove, while the hydrophilic moieties (e.g., carbohydrates and peptides), if present, extend out, and are thus exposed to TCRs. Most of the data on interactions of TCR with lipid antigens come from the studies on CD1d-restricted iNKT cells. Similarly to MHC-restricted T-cell response, CD1-restricted T-cell response requires recognition of the CD1-ligand complex by the TCR, and that contacts be made between the TCR binding site and both the CD1 molecule and the hydrophilic head of the lipid ligand (Borg et al. 2007; De Libero and Mori 2010a; Van Rhijn et al. 2013). One notable exception is activation of CD1a-restricted T cells by CD1a complexes with head-less hydrophobic lipids found in the sebum, in which case only the contact of the TCR with CD1a triggers the T cells (further discussed in the chapter “Endogenous lipids presented by CD1 molecules”) (de Jong et al. 2014). The strength of T-cell activation depends on the kinetics of TCR and CD1-ligand complex binding. Complexes that show the highest affinity to TCRs are the strongest agonists (Cantu et al. 2003; De Libero and Mori 2010a; McCarthy et al. 2007). All CD1 proteins contain binding pockets A′ and F′, so named after analogously located MHC Class I A and F pockets (Fig. 1). CD1b has the largest binding groove (2200 Å^3^), followed by CD1c (1780 Å^3^) and CD1d (1650 Å^3^), with CD1a groove being the smallest (1350 Å^3^) (Dellabona et al. 2015). CD1b also reveals two additional pockets: C′, corresponding to the C pocket in MHC I, and T′, which stands for the “tunnel” that connects A′ and F′ pockets (Ly and Moody 2014).

**Table 2:** Exogenous and endogenous lipid antigens presented by CD1 molecules

Source	Lipid antigen	CD1 isoform	References
*Mycobacterium tuberculosis*	Didehydroxymycobactins	CD1a	Moody et al. 2004
*M. tuberculosis* *Mycobacterium* spp	Mycolic acids	CD1b	Beckman et al. 1994
*M. tuberculosis* *Nocardia farcinica*	Glucose monomycolate	CD1b	Moody et al. 1997 Batuwangala et al. 2004
*Mycobacterium bovis* BCG *M. tuberculosis*	Glycerol monomycolate	CD1b	Layre et al. 2009
*M. tuberculosis*	Sulfoglycolipid Diacylated sulfoglycolipids	CD1b	Guiard et al. 2009 Gilleron et al. 2004
*M. tuberculosis* *Mycobacterium leprae*	Lipoarabinomannan	CD1b	Sieling et al. 1995
*M. tuberculosis*	Phosphatidylinnositol mannosides	CD1b CD1d	Cala-De Paepe et al. 2012 Fischer et al. 2004
*M. tuberculosis* *Mycobacterium avium*	Isoprenoid glycolipids Hexosyl-1-phosphoisoprenoids Mannosyl-*β*-1-phosphodolichols	CD1c	Moody et al. 2000
*Corynebacterium glutamicum* *M. tuberculosis* *Listeria monocytogenes*	Phospholipids Diphosphatidylglycerol Phosphatidylinositol Phosphatidylglycerol	CD1d	Fischer et al. 2004 Tatituri et al. 2013 Wolf et al. 2015
*Sphingomonas* spp *Ehrlichia muris*	Glycosphingolipids Glucuronosylceramide Galactouronosylceramide	CD1d	Kinjo et al. 2005 Mattner et al. 2005
*Bacteroides fragilis*	Glycosphingolipid	CD1d	Wieland Brown et al. 2013
*Borellia burgdorferi* *Streptococcus pneumoniae*	Diacylglycerols Galactosyl diacylglycerol Glucosyl diacylglycerol	CD1d	Kinjo et al. 2006 Kinjo et al. 2011
*Helicobacter pylori*	Glucosyl cholesterol	CD1d	Chang et al. 2011 Ito et al. 2013
*Aspergillus fumigatus*	Asperamide B *β*-linked glucosylceramide	CD1d	Cohen et al. 2011
*Leishmania donovani* *Entamoeba histolytica*	Lipophosphoglycans Acylated lysophosphatidyl inositol	CD1d	Amprey et al. 2004 Lotter et al. 2009
*Agelas mauritianus*	*α*-Galactosylceramide	CD1d	Kawano et al. 1997 Zajonc et al. 2005a Koch et al. 2005
*Cupressus sempervirens*	Phosphatidylcholine and phosphatidylethanolamine (18:2/18:2)	CD1a	Agea et al. 2005
*Toxicodendron* spp	Urushiol	CD1a	Kim et al. 2016
Mammalian (self)	Wax esters Triacylglycerols Free fatty acids Squalene	CD1a	de Jong et al. 2010 de Jong et al. 2014
	Sulfatide	CD1a, b, c CD1d	Zajonc et al. 2003 Shamshiev et al. 2002 Bai et al. 2012
	Isoglobotriaosylceramide (iGb3)	CD1d	Zhou et al. 2004b
	GM1 ganglioside	CD1b	Shamshiev et al. 2000
	GQ1b ganglioside	CD1b	Ishida et al. 1994
	GD3 ganglioside	CD1d	Wu et al. 2003
	Mannosyl *β*-1-phosphodolichol	CD1c	Moody et al. 2000
	Phospholipids and lysophospholipids Phosphatidic acid, phosphatidylinositol, phosphatidylethanolamine, phosphatidylcholine, phosphatidylglycerol, phosphatidylserine	CD1b, d	Gadola et al. 2002 Cox et al. 2009 Fox et al. 2009
	Sphingomyelin Phosphatidylcholine Phosphatidylinositol GM2 and GM3 gangliosides	CD1c, d CD1c, d CD1c CD1d	Haig et al. 2011
	Methyllysophosphatidic acids (leukemia cells)	CD1c	Lepore et al. 2014

### CD1a

CD1a is expressed on thymocytes and antigen-presenting cells, predominantly on epidermal Langerhans cells (Dougan et al. 2007). The small cleft of CD1a also reveals the simplest structure in comparison with all the other isoforms, limiting the size of bound lipids. Because of a clearly delineated path through the interior of the molecule, which abruptly terminates in the A′ pocket, CD1a has been described as a molecular ruler selecting alkyl chains of a defined length (Zajonc et al. 2003). The first identified exogenous lipids presented by CD1a were dideoxymycobactin lipopeptides, which belong to the mycobacterial siderophores family. Dideoxymycobactin antigens are composed of a single *N*-acyl chain, which fits in CD1a A′ pocket, and a complex peptide head group accommodated by F′ pocket (Fig. 1) (Moody et al. 2004; Rosat et al. 1999; Young et al. 2009; Zajonc et al. 2005b). Phosphatidylcholine and phosphatidylethanolamine that contain only linoleic acids (18:2/18:2), found in cypress pollen, were shown to stimulate proliferation of CD1a-restricted T cells from cypress allergic subjects (Agea et al. 2005). Since phospholipids and glycolipids make up over 50% of pollen grain membranes, and CD1a is frequently overexpressed in pulmonary dendritic cells and bronchoalveolar lavage suspensions from allergic individuals, it is suspected that these compounds play an important role in the pathogenesis of pollen allergy (Russano et al. 2008; Spinozzi and Porcelli 2007). Recently, CD1a has been demonstrated to recognize urushiol, a sap compound found in the plants of the genus *Toxicodendron*, which causes poison-ivy dermatitis, an inflammatory skin disease. The malady is triggered by interaction of urushiol with CD1a on Langerhans cells and driven by CD1a-restricted T cells. Among different urushiol isoforms, a pentadecylcatechol with two unsaturations (C15:2) turned out to be the dominant immunogen. Upon entry into the CD1a cleft, the aromatic catechol head group fills the A′ pocket, while the aliphatic tail spans the F′ pocket. About 80% of the molecule is buried within CD1a, with the remainder left exposed to TCR (Kim et al. 2016).

### CD1b

CD1b is expressed on thymocytes and dendritic cells (Dellabona et al. 2015). The largest binding groove of CD1b is also the most convoluted with its network of interconnected pockets and tunnels, which has been described as a maze for alkyl chains (Gadola et al. 2002a). This complex organization allows it to accommodate molecules containing long fatty acids (C70-80), such as mycolic acid and its derivatives: glucose monomycolate and glycerol monomycolate (Beckman et al. 1994; Gadola et al. 2002a). Shorter mycolic acids (C32–54), such as those found in *Corynebacterium* and *Nocardia* spp., may also be presented by CD1b (Huang et al. 2011), raising a question of how such relatively small compounds remain accessible for the TCR rather than collapse inside the CD1b cleft. Presumably, this is achieved due to endogenous lipids, which assume the role of scaffolds. In the absence of a foreign ligand, spacious CD1b may be stuffed with two or more endogenous lipids. Smaller foreign ligands eject the endogenous lipids from the top of the cleft and sit on the lipids located deeper (usually a diacylglyceride or deoxydihydroceramide), which lend the foreign lipids upward support. Large foreign lipids, capable of filling the entire CD1b cleft, may oust all scaffold lipids (Huang and Moody 2016; Van Rhijn et al. 2015). Interestingly, even the largest recognized lipids (C80) are loaded into CD1b intact, despite structural studies suggesting that such lipids are too bulky to fit in the cleft. Since lipids, unlike proteins, are not easily trimmed to fit the presenting molecule, anchoring of such large ligands is probably accomplished due to an additional (accessory) C′ portal, which acts as an escape hatch, through which oversized alkyl chains are pushed to the outer surface side of CD1b (Cheng et al. 2006).

The big groove of CD1b also allows presentation of the cell wall polymers synthesized by *Mycobacterium, Corynebacterium*, and *Rhodococcus*, such as phosphatidylinositol mannosides (PIM), lipomannans, and lipoarabinomannans (Fischer et al. 2004; Sieling et al. 1995).

**Fig. 2:**
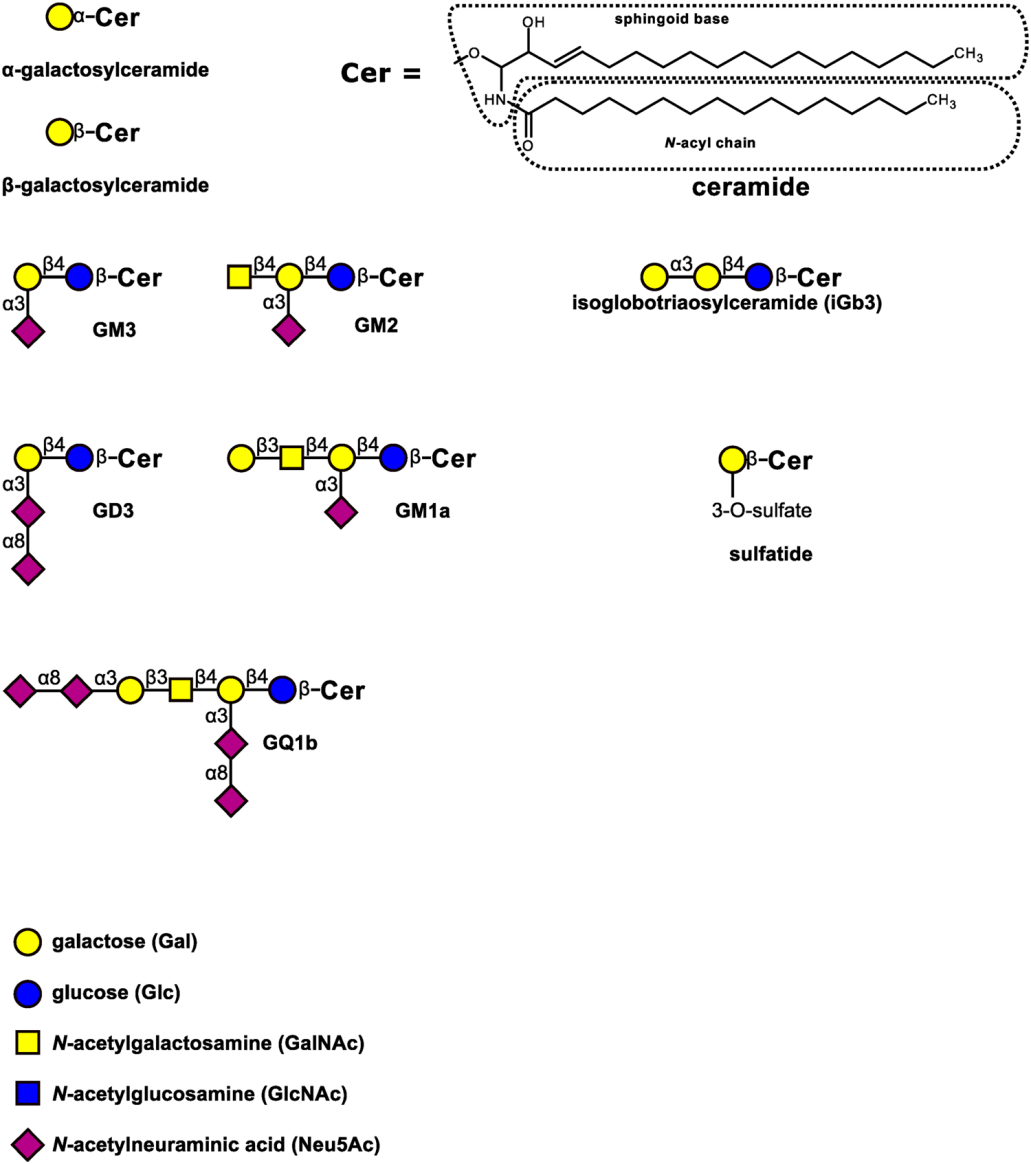
Schematic representation of example glycolipids recognized by CD1 molecules

CD1b also presents diacylated sulfoglycolipids expressed by *M. tuberculosis*, which consist of trehalose sulfate acylated at position 2 by short-chain fatty acids (predominantly C16:0 or C18:0) and at position 3 by hydroxyphtioceranoic acid, a long (C32) fatty acid with multiple methyl branches. Short-chain length and extravagant structure of the acyl chains at positions 2 and 3, respectively, dictate the antigenicity of these sulfoglycolipids, which fail to productively engage TCR when these chains are too long (position 2) or branchless (position 3). Correct configuration of chiral carbons in hydroxyphtioceranoic acid is also required to trigger T-cell response (Guiard et al. 2009). Similar to *Nocardia* short-chain mycolic acids, sulfoglycolipids that are not able to completely fill up the binding groove can be associated with scaffold lipids (Fig. 2).

### CD1c

CD1c is expressed on dendritic cells and Langerhans cells (where together with CD1a, it is one of the only two expressed isotypes), as well as on subsets of B cells (Adams 2013). In contrast to other CD1 isoforms, CD1c is riddled with three additional accessory portals: D′, E′, and F′ (Huang and Moody 2016; Scharf et al. 2010). As in the case of C′ portal of CD1b, these perforations either function as vents for oversized alkyl chains or additional points of entry. The A′ pocket in CD1c is larger than in other CD1 proteins and contains a large central pole, which creates a torque that coils an incoming alkyl chain around it (De Libero and Mori 2010b; Huang and Moody 2016). CD1c shows remarkable flexibility and may change its overall conformation from completely unroofed to shut upon binding of a ligand, reminiscent of a venus fly trap snapping over its prey (Mansour et al. 2016). CD1c binds mycobacterial phosphodolichols and phosphomycoketides (Brigl and Brenner 2004). Mycoketides are highly branched lipids, containing a single alkyl chain; they function as growth promoters of mycobacteria in host cells (Sirakova et al. 2003), and are produced by pathogenic strains of *Mycobacterium tuberculosis, Mycobacterium avium*, and *Mycobacterium bovis*, but not by fast-growing environmental mycobacteria. Studies on synthetic analogs of mannosyl phosphomycoketide revealed that the length, methyl branching pattern and the stereochemistry of the polyketide chain are essential features influencing the T-cell response (de Jong et al. 2007). Studies on phosphomycoketide-reactive T-cell clones isolated from peripheral blood mononuclear cells with phosphomycoketide-loaded CD1c showed that there are three types of reactivity of T cells: with mannosyl phosphomycoketide, phosphomycoketide, or both (Roy et al. 2014). It was shown that mycoketide antigens are bound in the A′ pocket and that methyl groups in the mycoketide chain (of C30–C34 length) are essential for loading into CD1c (Roy et al. 2014). The F′ pocket in CD1c is distinctively open to the solvent and its main role seems to be assistance in loading mycoketide chains into the A′ pocket. When smaller lipids are anchored, the F′ pocket may be filled with spacer lipids, named so to distinguish them from scaffold lipids, for the lack of upward lifting function in this case (De Libero and Mori 2010b). It was shown that infection with *Borrelia burgdorferi* causes upregulation of CD1c on myeloid dendritic cells, but the ligands are yet to be determined. In silico studies suggest that acylated steryl glycosides from *B. burgdorferi* may be loaded into CD1c (Mansour et al. 2016; Yakimchuk et al. 2011). In addition, CD1c was found to present synthetic lipopeptide acylated at N-terminus (Van Rhijn et al. 2009). Interestingly, this ability is lost when CD1c is rerouted to lysosomes, which underscores the importance of traversing different intracellular compartments by different CD1 proteins for ability to recognize and present their typical ligands (Adams 2013; Van Rhijn et al. 2009).

### CD1d

In contrast to CD1 proteins belonging to group 1, CD1d is present on many cell types, including dendritic cells, B cells, monocytes, macrophages, keratinocytes, and gastrointestinal epithelial cells (Brigl and Brenner 2004). In terms of structure complexity and binding capacity, CD1d may be considered intermediate when compared with other CD1 molecules. Its binding groove contains clearly defined A′ and F′ pocket with no accessory portals (Salio et al. 2014). The toroidal A′ pocket of CD1d is closed and allows the insertion of alkyl chains with a defined length, while the F′ pocket contains a closed roof that makes binding of very long lipid chains impossible (De Libero and Mori 2010a).

The first discovered and most extensively studied ligand for CD1d is *α*-galactosylceramide (*α*GalCer), a glycosphingolipid isolated from marine sponge *Agelas mauritianus* (Fig. 2) (Koch et al. 2005; Zajonc et al. 2005a). The F′ and A′ pockets of CD1d can perfectly accommodate the C18 sphingosine chain and C26 acyl chain of *α*GalCer, respectively. The galactose ring extends out of the groove and is thus exposed for interaction with TCR expressed on iNKT cells (Tsuji 2006). There are other, structurally similar glycosphingolipids, that are presented by CD1d, such as αGalCer from *Bacteroides fragilis* (a commensal of human and mouse gut microbiota), *α*-glucuronosylceramide from *Sphingomonas capsulata, α*-galacturonosylceramide from *Sphingomonas yanoikuyae* and *Sphingomonas wittichii, α*-galactosyldiacylglycerol from *B. burgdorferii*, and glycosylated diacylglycerols from *Streptococcus pneumoniae* (Ben-Menachem et al. 2003; Kinjo et al. 2006, 2011; Tsuji 2006; Wieland Brown et al. 2013).

Similar to CD1b, the CD1d also binds PIM, a lipid synthesized by mycobacteria (Fischer et al. 2004). Natural PIMs derived from *Mycobacteria* may contain one to six mannose residues linked to acylated phosphatidylinositol. The PIM found in *M. bovis* contains four mannose residues and is, therefore, called PIM4 (Fischer et al. 2004). CD1d was also found to recognize phospholipids from *Corynebacterium glutamicum* or *M. tuberculosis*, including phosphatidylglycerol, diphosphatidylglycerol (cardiolipin), and phosphatidylinositol (Fischer et al. 2004; Tatituri et al. 2013). All were shown to stimulate type II NKT cells, but not iNKT cells, and thereby trigger antigen-specific interferon (IFN)-*γ* production and cell-mediated cytotoxicity. In addition, diphosphatidylglycerol of both bacterial and mammalian origin was shown to stimulate *γδ* T cells, which in response secrete IFN-*γ* and RANTES, and could thus function as sentinels during infection and tissue injury (Dieudé et al. 2011).

CD1d was also shown to recognize *α*-galactosyl diacylglycerolipids found in *B. burgdorferii* and *S. pneumoniae*, the causative agents of Lyme disease and pneumonia, respectively (Kinjo et al. 2011). It was demonstrated that iNKT cells are activated in vivo during an infection with *B. burgdorferii*; this step was shown to be crucial for the prevention of chronic joint inflammation and spirochete clearance (Tupin et al. 2008). Small changes in length and saturation of alkyl chains strongly influence the antigenic potency of the lipids (Kinjo et al. 2006). Similarly, recognition of antigens from *S. pneumoniae* was shown to rely on the presence of 18:1 vaccenic acid linked to sn-2 carbon in glycerol residue and on the position of a single unsaturation (Kinjo et al. 2011). Thus, it seems that the requirements which the ligands must fulfill to be recognized by CD1d go beyond the anomeric configuration of the sugar and are quite stringent.

Glycolipids structurally related to cholesterol, derived from *Helicobacter pylori*, such as cholesteryl 6-tetradecanoyl-*α*-glucoside, were also identified as NKT cell stimulants (Chang et al. 2011).

Although the majority of exogenous lipids recognized by CD1d are derived from bacteria, there are also known CD1d-presented antigens of protozoan and fungal origin. Protozoan surface glycoconjugates such as *Leishmania donovani* lipophosphoglycans (Amprey et al. 2004), and *Entamoeba histolytica* lipopeptidophosphoglycans (Lotter et al. 2009) were shown to stimulate iNKT cells and thus stifle the development of amebic liver abscess. Asperamide B, *β*-glucosylceramide derived from *Aspergillus fumigatus*, is the first known fungal glycolipid capable of iNKT cell activation in a CD1d-dependent manner (Albacker et al. 2013).

### CD1e

In contrast to CD1a-d, CD1e is a soluble intracellular protein (Facciotti et al. 2011; Garcia-Alles et al. 2011). It is expressed only in dendritic cells and resides in late endosomal and lysosomal compartments, where it assists in lipid editing and loading onto presenting CD1 molecules. It has been found to assist in turning PIM6 from *M. tuberculosis* into PIM2 motif for presentation by CD1b and in trimming of carbohydrate moieties of glycolipids (Cala-De Paepe et al. 2012).

## Endogenous Lipids Presented by CD1 Molecules

Endogenous lipids, such as phospholipids, lysophospholipids, plasmalogenes, sulfatide, or gangliosides, can be recognized by different CD1 molecules (Cox et al. 2009; Dellabona et al. 2015). The majority of endogenous lipids can be presented by all CD1 isoforms, but some isoforms reveal preference to one or more lipid types (Dellabona et al. 2015). One example of such lipids is sulfatide, a sulfoglycolipid found mainly in the myelin sheath. It is bound by all types of CD1 isoforms, but binding of CD1a is the most stable (Shamshiev et al. 2002; Zajonc et al. 2003). Gangliosides, which are glycosphingolipids containing one or more sialic acid residues, are also recognized by CD1 molecules. The most abundant ganglioside in the human myelin sheath, GM1 (GM1a, II^3^-*α*-Neu5Ac-Gg4Cer), is bound by CD1b (Chester 1997; Shamshiev et al. 2000), and so is GQ1b, which is also present in human brain and contains the same sugar root structure as GM1, but four sialic acid residues instead of one (Ishida et al. 1994) (Fig. 2). Since CD1b is expressed by cells in areas of demyelination in multiple sclerosis, it has been proposed that gangliosides and probably other self-lipids derived from myelin debris, such as fast-migrating cerebrosides, may be presented by astrocytes and microglia, and thus propel a vicious cycle of chronic inflammation (Gately et al. 2013; Shamshiev et al. 1999). Involvement of CD1d-restricted iNKT cells in multiple sclerosis is more complicated and seems immunoregulatory rather than exacerbatory (Hogan et al. 2013).

The carbohydrate moiety of most endogenous glycolipids is *β*-linked to the lipid portion, while exogenous glycolipids typically contain *α*-glycosidic linkage (Cheng et al. 2011). Indeed, it was long thought that *α*-glycosylceramides are not produced by mammalian cells at all. This conviction prompted a perennial search for the glycolipid self-antigen responsible for the positive selection of iNKT cells (which strongly respond to *α*-glycosylceramides) in the thymus. Several studies explored isoglobotriaosylceramide (iGb3) as the selecting self-antigen, but their conflicting results failed to provide the conclusive evidence (Facciotti et al. 2012; Godfrey et al. 2006; Porubsky et al. 2007, 2012; Speak et al. 2007; Yu et al. 2011; Zhou et al. 2004a) (Fig. 2). The most dubious aspect of these studies was the relevance of iGb3 in humans, because the human iGb3 synthase gene (*A3GALT2*) is inactive (Christiansen et al. 2008; Lawson 2012; Sanderson et al. 2013). Surprisingly, it was later demonstrated that mammalian immune cells indeed produce small quantities of *α*-glycosylceramides, contrary to the long-standing notion (Kain et al. 2014). Thus, the important question is: how are these glycolipids synthesized? Since there is no obvious glycosyltransferase gene candidate, we suggest that *α*-galactosylceramide in humans may be a secondary product of a promiscuous enzyme. Examples of such unfaithful glycosyltransferases are known (Kaczmarek et al. 2014, 2016; Suchanowska et al. 2012; Togayachi et al. 2001; Westman et al. 2015). Typically, in comparison to the main enzyme products, such secondary products occur in minute quantities, which may be unmeasurable when using classical biochemical methods, yet important in biological environment (Kain et al. 2014, 2015). Thus, iNKT cell selection is “more than meets the eye”, while the elusive self-antigen could arise as a result of biological parsimony.

Other endogenous lipids recognized by CD1d include phosphatidic acid, phosphatidylinositol, phosphatidylethanolamine, phosphatidylcholine, phosphatidylglycerol and phosphatidylserine, and their lyso-derivatives. Plasmalogens and cardiolipins were also shown to be recognized by CD1d (Cox et al. 2009).

Interestingly, components of human skin sebum, i.e., wax esters, triacylglycerides, free fatty acids, and squalene, were identified as CD1a-presented antigens (de Jong et al. 2010, 2014). These lipids are unusual CD1 ligands, because they do not contain a hydrophilic head group, so their nature is completely hydrophobic. It has been proposed that they nestle deep inside the CD1a groove and induce the correct conformation of CD1a for direct interaction with the TCR, which may lead to T-cell activation. Recognition of these lipids occurs only upon disruption of the skin barrier, and thus alerts the immune system to the danger before invasion of microbes ensues. Without such challenge, CD1a binds ubiquitous amphipathic non-stimulatory lipids (e.g., phospholipids), which need to be replaced by hydrophobic head-less lipids for T cell activation (de Jong 2015). CD1a was also shown to recognize lipid products of phospholipases secreted by house dust mites, which thus could be implicated in atopic dermatitis (Jarrett and Ogg 2016).

## Anti-lipid Antibodies

**Fig. 3:**
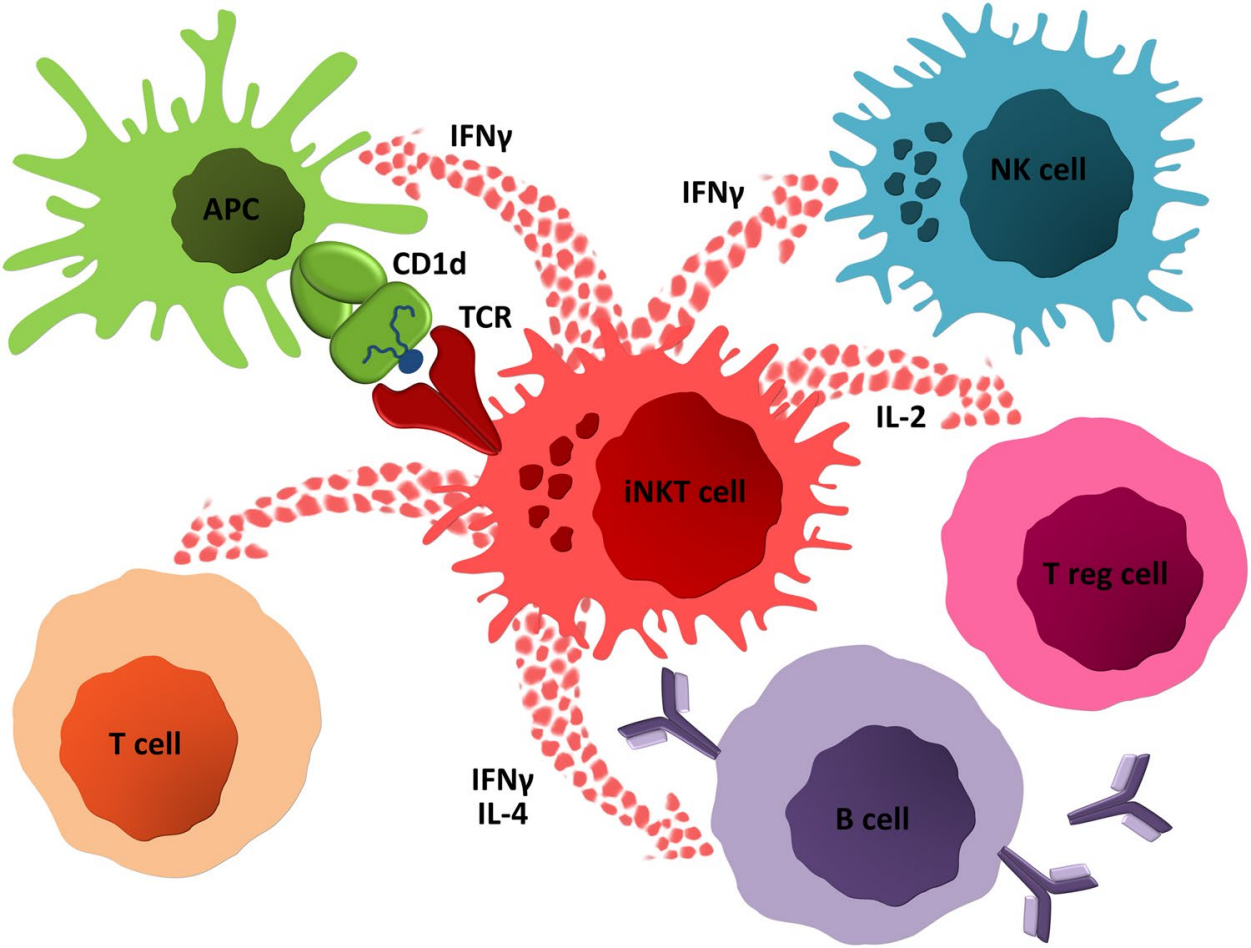
Activated iNKT cells may rapidly secrete high amounts of both T_H_1 and T_H_2-type cytokines, and so regulate and activate many different cell types

**Fig. 4:**
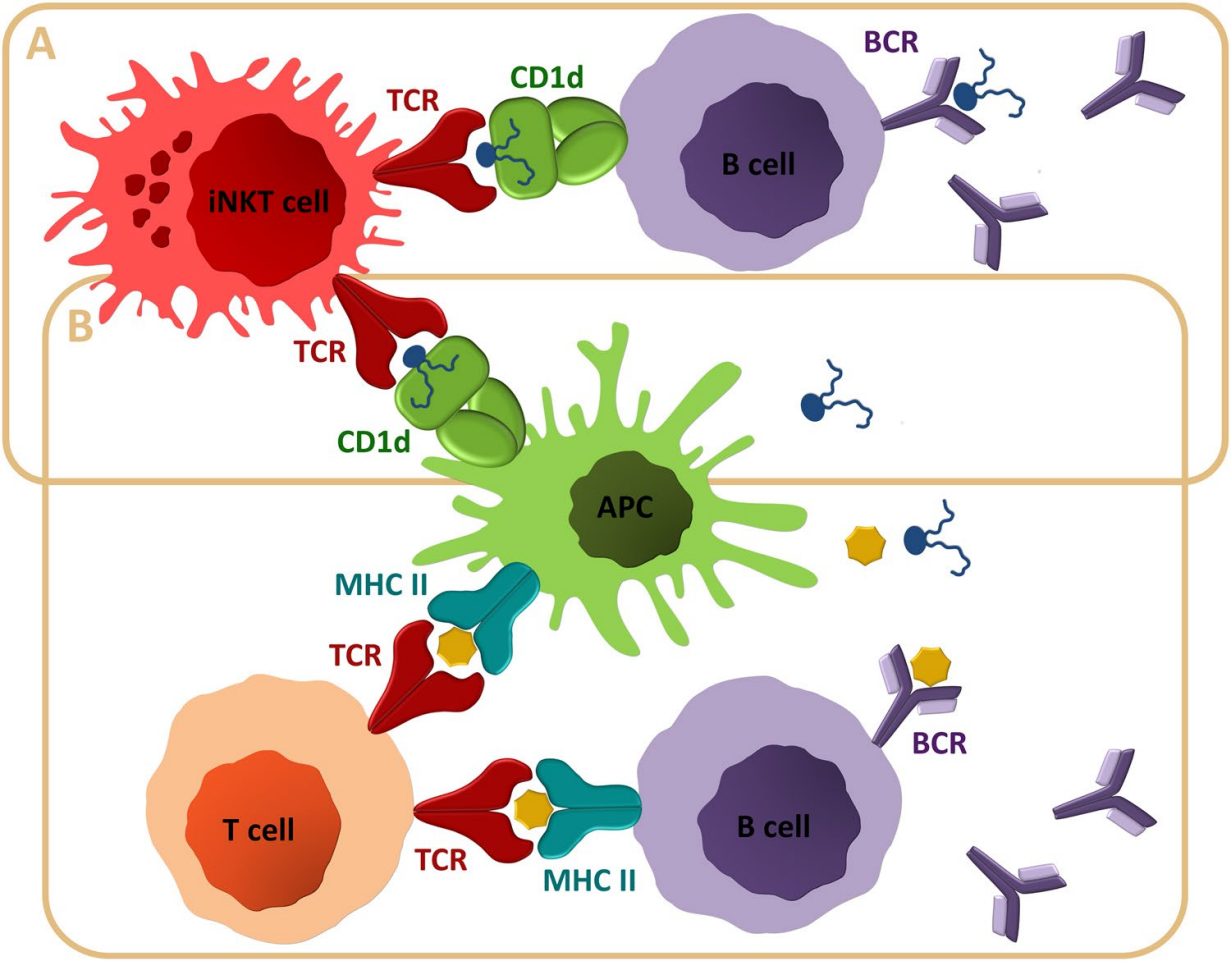
iNKT cells can provide cognate and non-cognate T cell help to B cells. **a** Cognate help is characterized by rapid and transient anti-lipid antibody production, formation of short-lived germinal centers, but no generation of memory cells. **b** Non-cognate help of iNKT to B cells arises when peptide antigen is admixed with *α*GalCer (or other iNKT agonist). Antigen-presenting cells are able to simultaneously present peptide antigens on MHC Class II and *α*GalCer on CD1d. Upon recognizing *α*GalCer-CD1d complex, activated iNKT cells promote presentation of peptide antigens to T cells. Subsequent interaction between T and B cells proceeds like a typical T-cell-dependent response, resulting in generation of antibodies against peptide antigen. The non-cognate iNKT cell help is independent of CD1d expression on B cells

Anti-lipid antibodies arise in a number of infectious and autoimmune diseases, including leprosy, tuberculosis, systemic lupus erythematosus (SLE), or occur naturally. One example of such antibodies is the ones that recognize blood group antigens, which may be detrimental upon mismatched blood transfusion. However, despite these prominent roles, little data are available on the mechanism of emergence of anti-lipid antibodies (King et al. 2011; Poole and Daniels 2007; Wong-Baeza et al. 2016). Most studies on the role of CD1 system in immune response focus on CD1d-restricted iNKT cells, because these cells represent a relatively clearly defined CD1-restricted T-cell subpopulation. Yet, activated iNKT cells may rapidly secrete high amounts of both T_H_1 and T_H_2-type cytokines, and so regulate and activate a myriad of different cell types (macrophages, dendritic cells, and B and T cells) (Fig. 3). Thus, iNKT cell activation may lead to opposite outcomes, causing amelioration in some pathologies, but exacerbating others (Crosby and Kronenberg 2016; King et al. 2011; Novak et al. 2013; Salio et al. 2014; Siegmann et al. 2014). iNKT cells may provide both non-cognate (bystander) and cognate T cell help to B cells. The non-cognate help is more prominent, so iNKT agonists (primarily α-galactosylceramide) have been utilized as adjuvants (Artiaga et al. 2016; Cerundolo et al. 2009; Crosby and Kronenberg 2016; Galli et al. 2007; Novak and Lehuen 2011). In contrast, cognate help for lipid-specific B cells attracted lesser attention. Yet, anti-lipid antibodies of IgG and IgM classes have been described (Poole and Daniels 2007; Wong-Baeza et al. 2016). IgM antibodies may arise against glycoglycerolipids of symbiotic lactobacilli in inflammatory bowel disease (Iwamori et al. 2011; Paściak et al. 2016). Actinobacterial glycoglycerolipids and saccharolipids also show antigenicity (Paściak et al. 2003, 2004, 2010). A synthetic haptenated *α*-galactosylceramide was shown to induce robust primary IgG antibodies through cognate help from iNKT cells to B cells, but the response wanes quickly and is not followed by memory development or long-lived plasma-cell differentiation (King et al. 2011; Leadbetter et al. 2008) (Fig. 4a). In contrast, non-cognate iNKT cell help for protein-reactive B cells leads to memory and plasma-cell development, and long-lived antibody responses (King et al. 2011) (Fig. 4b). Anti-glycolipid antibodies usually recognize epitopes localized on the carbohydrate moieties of glycolipids. Still, because of their lipid nature, glycolipids are believed to elicit humoral response through CD1d presentation to iNKT cells and their cognate help to B cells. Perplexingly, this may not always be the case, because anti-iGb3 and anti-B blood group antigen antibodies were shown to arise in CD4^+^ T-cell-dependent, but CD1d and iNKT cell-independent manner (Christiansen et al. 2011); the exact mechanism, though, remains obscure. In contrast, invariant TCR-CD1d interaction was shown to be necessary for the emergence of antibodies against A blood group antigen (Tazawa et al. 2013). Since glycolipids carrying A and B blood group antigens differ only by *N*-acetamide moiety in the terminal sugar residue (*N*-acetylgalactosamine in A versus galactose in B blood group antigen), it is striking that such a small difference may determine which part of the immune system is involved in response.

Many anti-lipid (often anti-glycolipid) antibodies are hallmarks of autoimmune diseases, such as type 1 diabetes mellitus, multiple sclerosis, autoimmune hemolytic anemias (Ii blood group system-related cold agglutinin disease and paroxysmal cold hemoglobinuria), and SLE (Bovin et al. 2012; Kaczmarek et al. 2014). Despite the prominent role of CD1-restricted cells in general and iNKT cells in particular in these pathologies, the link between these cells and the emergence of anti-lipid autoantibodies has rarely been investigated (Bovin et al. 2012; Novak and Lehuen 2011). IgG autoantibodies found in SLE, which recognize mainly phospholipid self-antigens, were shown to arise as a result of double-negative T-cell help in a CD1c-restricted manner (Sieling et al. 2000). Recently, it has been demonstrated that success or failure of iNKT cells to control self-reactive B-cell responses is driven by their interaction with neutrophils during inflammation (Hägglöf et al. 2016). Since B-cell response prompted by cognate iNKT cell help is rapid but transient, it could paradoxically be viewed as an element of innate response to danger, despite the humoral component. This is, perhaps, the most vivid example of how CD1 presentation system bridges the innate and adaptive immune response (Lawson 2012).

## Concluding Remarks

CD1 system continues to draw attention despite its complexity and ambiguities. The rapid nature of immune response involving CD1 system drives interest to exploit it in vaccination strategies, as well as in infection and autoimmunity control. One feature in particular makes CD1 proteins interesting targets for drug design: in stark contrast to highly donor-restricted patterns of MHC–TCR interaction, they are non-polymorphic in human populations. Thus, CD1–TCR interactions may be described as donor-unrestricted, which raises the prospect that lipid agonists and antagonists of T cells could be developed (Huang and Moody 2016; Van Rhijn and Moody 2015). This, however, will require a complete characterization of CD1-restricted cell subpopulations, CD1 ligand-binding profiles, and thorough understanding of all factors that contribute to the outcome of CD1-restricted cell stimulation.
